# Draft Genome Sequence of *Burkholderia sordidicola* S170, a Potential Plant Growth Promoter Isolated from Coniferous Forest Soil in the Czech Republic

**DOI:** 10.1128/genomeA.00810-14

**Published:** 2014-08-14

**Authors:** Salvador Lladó, Zhuofei Xu, Søren J. Sørensen, Petr Baldrian

**Affiliations:** a Laboratory of Environmental Microbiology, Institute of Microbiology of the ASCR, v.v.i., Praha, Czech Republic; b Section of Microbiology, Department of Biology, University of Copenhagen, Copenhagen, Denmark

## Abstract

*Burkholderia* species are key players in the accumulation of carbon from cellulose decomposition in coniferous forest ecosystems. We report here the draft genome of *Burkholderia sordidicola* strain S170, containing features associated with known genes involved in plant growth promotion, the biological control of plant diseases, and green remediation technologies.

## GENOME ANNOUNCEMENT

*Burkholderia sordidicola* is a Gram-negative, nonmotile, non-spore-forming, ovoid- to rod-shaped bacterium. It was first isolated from the white rot fungus *Phanerochaete sordida* (1) and later found in the rhizosphere (2) and mycosphere of soils (3), plant roots (4), and root nodules (5).

Many *Burkholderia* species are well known for their metabolic capabilities, which can be exploited for biotechnological purposes, such as the biological control of plant diseases (6), nodulation and plant growth promotion (7), and bioremediation (8).

*B. sordidicola* strain S170 was isolated from the topsoil of an unmanaged acidic coniferous forest dominated by spruce (*Picea abies*). Studies on the active bacterial communities involved in important biogeochemical processes in this soil found that *Burkholderia* was one of the most abundant genera (9). Moreover, based on stable isotope-probing members of the genus *Burkholderia* are among the most abundant bacteria that accumulate C from cellulose and likely contribute to its decomposition in this environment (10).

The draft genome was sequenced on an Illumina MiSeq platform via a paired-end run (2 × 251 bp). This run yielded 2,024,618 reads, representing approximately 42-fold coverage. The sequence data were assembled using SPAdes 3.0 (11), generating 132 contigs >500 bp (*N*_50_, 208,852 bp) that represented the *B. sordidicola* strain S170 genome. Gene calling and annotation were performed using Rapid Annotations using Subsystems Technology (RAST) 4.0. The total combined contig size is 10,273,854 bp, containing 9,904 coding sequences (CDSs), of which 66.4% were assigned to known functional genes.

Of the genes that were assigned, 1-aminocyclopropane-1-carboxylate (ACC) deaminase activity and proteins NodN and NodD may potentially contribute to plant growth promotion and the nodulation activity of strain S170, respectively. No nitrogen fixation-coding genes were found. Several predicted genes involved in iron acquisition and metabolism (*n* = 13) and phosphorous metabolism (*n* = 71), including the PHO operon to assimilate inorganic phosphate, and the lack of toxin-coding genes suggest that strain S170 may be a good candidate for biofertilizer trials. The annotated genome also revealed many genes involved in the metabolism of aromatic compounds (*n* = 241) and of carbohydrates (*n* = 964). Furthermore, analysis of the genome using dbCAN (12) showed that the strain S170 genome contains 282 genes coding for different carbohydrate-active enzymes (CAZY), with 64 glycoside hydrolases (GH) among them (e.g., β-glucosidase, cellulose, and other enzymes related to cellulose and other polysaccharide decomposition). This capability to use many different C sources in combination with several putative ABC transporters make strain S170 a strong candidate for bioaugmentation in bioremediation technologies and may potentially allow it to colonize many different plant niches. Several genes for the biosynthesis of bacteriocins (*n* = 11) and resistance to antibiotics and toxic compounds (*n* = 178) were also found. The presence of these genes in combination with the above-mentioned lack of toxin-coding genes suggests that *B. sordidicola* strain S170 may also be a suitable candidate for the biological control of diseases in plants.

### Nucleotide sequence accession numbers.

The draft sequence of *B. sordidicola* S170 was deposited at DDBJ/EMBL/GenBank under the accession number JNFG00000000. The version described in this paper is version JNFG01000000.
